# Dietary Ellagic Acid Mitigates High Stocking Density-Induced Oxidative Stress and Immune Suppression in Common Carp (*Cyprinus carpio*): An In Vivo and In Silico Study

**DOI:** 10.3390/antiox15081012

**Published:** 2026-08-13

**Authors:** Serpil Mişe Yonar, Mücahit Eroğlu, Mehmet Nuri Cakmak, Harun Uslu, Mevlüt Şener Ural, Cemal Orhan, Muhammet Enis Yonar

**Affiliations:** 1 Department of Aquaculture, Fisheries Faculty, Firat University, Elazig 23200, Türkiye; s_mise@firat.edu.tr (S.M.Y.); mncakmak@firat.edu.tr (M.N.C.); msural@firat.edu.tr (M.Ş.U.); 2 Department of Basic Sciences, Fisheries Faculty, Firat University, Elazig 23200, Türkiye; meroglu44@firat.edu.tr; 3 Department of Pharmaceutical Chemistry, Faculty of Pharmacy, Firat University, Elazig 23200, Türkiye; huslu@firat.edu.tr; 4 Department of Animal Nutrition, Faculty of Veterinary Medicine, Firat University, Elazig 23200, Türkiye; corhan@firat.edu.tr

**Keywords:** ellagic acid, immune response, molecular docking, oxidative stress, stocking density

## Abstract

This study examined the effects of dietary ellagic acid (EA) on growth performance, survival, immune responses, and oxidative status of common carp (*Cyprinus carpio*) exposed to high stocking density (HSD). A 2 × 3 factorial design was used, with two stocking densities (25 and 100 kg/m^3^) and three EA levels (0, 50, and 100 mg/kg diet). HSD significantly reduced growth performance, as indicated by lower weight gain and specific growth rate (SGR), increased feed conversion ratio (FCR), and decreased survival (*p* < 0.05). It also suppressed immune responses, including white blood cell count (WBC), nitroblue tetrazolium activity (NBT), phagocytic activity (PA), lysozyme activity (LYZ), and bactericidal activity (BA). Furthermore, HSD increased malondialdehyde High stocking density caused (MDA) levels and decreased superoxide dismutase (SOD), catalase (CAT), and glutathione peroxidase (GSH-Px) activities in liver, head kidney, and spleen tissues (*p* < 0.05). Fish were challenged by intraperitoneal injection of *Aeromonas salmonicida* subsp. *achromogenes*. Dietary EA supplementation significantly improved survival under both stocking densities, with the highest survival observed in fish receiving 100 mg/kg EA. Kaplan–Meier survival analysis and log-rank tests confirmed significant differences among treatment groups (*p* < 0.05). Pairwise comparisons further demonstrated that EA, particularly at 100 mg/kg, enhanced survival under high-density conditions. EA supplementation also improved growth performance, antioxidant status, and immune responses in a dose-dependent manner. Polynomial contrast analysis revealed significant linear trends: increasing EA levels enhanced weight gain, antioxidant enzyme activities, and immune parameters, while reducing MDA levels (*p* < 0.05). Significant SD × EA interactions indicated that EA alleviated the adverse effects of crowding stress. In silico analyses showed higher binding affinity of EA toward SOD, CAT, and GSH-Px. Overall, dietary EA effectively mitigated density-induced oxidative stress and immunosuppression, supporting its potential as a functional feed additive in intensive aquaculture.

## 1. Introduction

Aquaculture is essential for meeting global demand for animal protein; however, scaling up often harms fish welfare. One of the most harmful environmental and management stressors is high stocking density (HSD), which has been repeatedly linked to poor growth, weakened immune responses, and lower survival rates across various fish species [1,2,3,4,5]. Under HSD conditions, fish are continuously exposed to social and environmental stress, which consistently activates the hypothalamic–pituitary–interrenal (HPI) axis, elevating cortisol levels and causing metabolic disruptions [6,7]. These hormonal imbalances result in higher plasma glucose levels, increased lipid peroxidation, weakened antioxidant defenses, and suppressed immune function-factors that collectively reduce aquaculture productivity and sustainability [8].

The common carp (*Cyprinus carpio*), one of the most widely farmed freshwater species worldwide, is particularly vulnerable to crowding stress given its economic importance and the widespread practice of intensive cultivation. Numerous studies have shown that stocking densities above roughly 80–100 kg/m^3^ significantly increase oxidative stress markers, alter hematological parameters, and cause tissue damage in vital organs such as the liver, kidney, and gills [9,10]. Therefore, there is a strong need to develop nutritional or husbandry strategies to mitigate these harmful effects and promote sustainable aquaculture.

Recently, research has focused on natural dietary antioxidants as safe, sustainable strategies to mitigate stress-related challenges in aquaculture [11,12]. Among these bioactive compounds, ellagic acid (EA), a polyphenol found in pomegranates, walnuts, raspberries, and other plants, has attracted significant attention. EA exhibits potent antioxidant, anti-inflammatory, and anti-apoptotic effects through mechanisms that include scavenging reactive oxygen species, preventing lipid peroxidation, and modulating key cellular signaling pathways such as Nrf2/ARE and NF-κB [13,14,15]. Most previous studies investigating the protective effects of EA in fish have focused on chemically induced oxidative stress models, including exposure to pesticides and heavy metals. Although these studies clearly demonstrated the antioxidant and cytoprotective properties of EA, chemically induced toxicity and husbandry-related chronic stress are biologically distinct. Unlike chemical toxicants, chronic crowding stress results from sustained activation of the HPI axis, persistent cortisol secretion, altered energy allocation, social interactions, and prolonged immune modulation. Consequently, the physiological mechanisms underlying density-induced stress cannot be simply extrapolated from chemically induced oxidative stress models, highlighting the need to evaluate EA under intensive aquaculture conditions [16,17].

In aquatic animals, EA has shown significant protective effects against environmental and chemical stressors. For example, Wang et al. [18] demonstrated that dietary EA supplementation alleviated chronic ammonia stress in yellow catfish by improving growth performance, reducing oxidative damage, enhancing antioxidant capacity, and promoting cellular protective mechanisms. Likewise, Yonar et al. [19] found that EA supplementation reduced chlorpyrifos-induced oxidative stress and immune suppression in carp, highlighting its potential as a natural therapeutic agent in aquaculture. Despite this promising evidence, the role of EA in mitigating HSD-induced stress has not yet been studied in common carp. Furthermore, unlike previous studies that primarily evaluated oxidative biomarkers, the present work integrates physiological performance, innate immunity, pathogen resistance, and in silico protein–ligand interactions to provide a broader understanding of the mechanisms by which EA supports fish health under intensive culture conditions.

As aquaculture intensifies, identifying nutritional strategies to improve fish resilience under chronic husbandry stress has become increasingly important. To our knowledge, no previous study has comprehensively evaluated the effects of dietary EA against HSD-induced stress in common carp by integrating growth performance, hematological and immune responses, bacterial disease resistance, oxidative stress biomarkers, and molecular docking analyses. Therefore, the present study extends current knowledge beyond previous chemically induced oxidative stress models by investigating whether EA can preserve physiological homeostasis under chronic crowding stress, a major welfare and sustainability challenge in modern aquaculture. We hypothesized that dietary EA supplementation would enhance antioxidant defenses, improve immune competence, increase resistance to bacterial infection, and ultimately improve the physiological resilience of common carp exposed to HSD.

## 2. Materials and Methods

### 2.1. Experimental Design

In this study, six experimental groups were established to evaluate the effects of dietary EA supplementation on oxidative stress and immune responses in common carp (*Cyprinus carpio*) exposed to different stocking densities.

All experimental procedures involving fish were conducted in accordance with the guidelines for the care and use of laboratory animals and were approved by the Fırat University Local Animal Experiments Ethics Committee (Approval No: 2025/12-06, Date: 8 July 2025). All efforts were made to minimize animal suffering and to reduce the number of animals used in the experiment.

A 2 × 3 factorial setup was used, involving two stocking densities (25 and 100 kg/m^3^) and three dietary EA levels (0, 50, and 100 mg/kg feed). Each treatment was replicated three times. Before the start of the experiment, fish were randomly allocated to 100 L experimental tanks while maintaining the predetermined stocking densities, resulting in 50 fish per tank for the 25 kg/m^3^ groups and 200 fish per tank for the 100 kg/m^3^ groups, with an initial average body weight of 50 ± 5 g. Blinding of investigators during feeding and sample collection was not feasible because of the nature of the experimental design. However, all laboratory analyses were performed using standardized protocols applied equally to all experimental groups. This setup enabled the examination of both the individual and combined effects of stocking density (SD) and dietary EA supplementation on oxidative stress and immune responses in common carp (*Cyprinus carpio*).

Water quality parameters were monitored regularly throughout the experimental period and maintained within the optimal range for common carp culture. Water temperature was maintained at 25 ± 1 °C, dissolved oxygen at 7.2 ± 0.5 mg/L, pH at 7.2 ± 0.2, and total alkalinity at approximately 120 mg CaCO_3_/L. These conditions were maintained throughout the experimental period to ensure a suitable rearing environment. Partial water exchange was performed regularly to maintain stable water quality and ensure suitable rearing conditions throughout the experiment.

### 2.2. Diet Preparation

EA, which is poorly soluble in water under natural conditions, was first dissolved in 70% ethanol to ensure homogeneous mixing with the basal feed. The required amounts of EA solution were sprayed onto a commercial pellet diet (Cagatay Oil and Feed Products Inc., Izmir, Türkiye; Ecobio, trout grower feed; proximate composition: 45% crude protein, 20% crude fat, 11% ash, 3% crude fiber, 8.5% moisture, 12.5% nitrogen-free extract, and 5124 kcal/kg gross energy). The control diet (0 mg/kg EA) was prepared using the same procedure, except that an equivalent volume of 70% ethanol without EA was sprayed onto the feed. After thorough mixing, all diets were spread in thin layers and air-dried at room temperature for 48 h to facilitate the evaporation of ethanol before storage and use. Residual ethanol concentration in the experimental diets was not analytically determined. Experimental diets were formulated to contain 0 (control), 50 (EA-50), and 100 (EA-100) mg EA per kg of feed, consistent with the factorial design of the study (2 × 3; two stocking densities × three dietary EA doses). Dried feeds were subsequently stored in airtight dark glass containers at 4 °C until use.

The commercial trout grower feed was selected as a standardized basal diet for all experimental groups. Since all fish received the same basal diet, EA supplementation was the only dietary variable among treatments, allowing the observed effects to be attributed specifically to ellagic acid rather than differences in the nutritional composition of the basal diet.

The inclusion levels of EA were selected based on previous studies demonstrating its protective effects against oxidative stress and immunosuppression in fish, with slight adjustments to align with the experimental design [13,19]. The selected EA supplementation levels (50 and 100 mg/kg diet) were chosen to represent biologically relevant low and moderate dietary supplementation levels based on previous studies demonstrating their antioxidant and immunomodulatory efficacy without adverse effects. The inclusion of two supplementation levels also enabled evaluation of a potential dose-dependent response under both low- and high-stocking-density conditions.

Stocking densities were determined by dividing total biomass by water volume, using the following formula:

SD (kg/m^3^) = Total fish weight (kg)/Tank volume (m^3^)

Each tank had a volume of 100 L (0.1 m^3^), and the average initial body weight of the carp was 50 ± 5 g (0.05 kg). Using this formula, the number of fish per tank was calculated to be 50 for the 25 kg/m^3^ groups and 200 for the 100 kg/m^3^ groups. Therefore, six experimental groups (2 stocking densities × 3 EA levels) were created, each in triplicate, resulting in a total of 18 tanks and 2250 fish. This method ensured that the selected stocking densities represented both commercially relevant low-density and intensive high-density culture conditions. The 25 kg/m^3^ treatment reflects stocking densities commonly used under optimal culture conditions, whereas 100 kg/m^3^ represents an intensive stocking density that can induce chronic crowding stress in tank-based aquaculture systems, consistent with previous studies on *Cyprinus carpio* and other freshwater species [1,20].

The feeding trial lasted for 12 weeks. During this period, fish were hand-fed to apparent satiation twice daily at 09:00 and 17:00. After each feeding, uneaten pellets were carefully removed to maintain water quality, and the daily feed intake was monitored throughout the experiment. To evaluate growth performance and feed utilization, individual fish body weights were measured weekly.

### 2.3. Sample Collection

At the end of the 12-week feeding period, thirty fish from each tank were counted and weighed to determine growth performance parameters, including weight gain (WG), specific growth rate (SGR), feed conversion ratio (FCR), and survival rate. Subsequently, randomly selected fish were anesthetized using benzocaine solution (25 mg/L), and blood samples were collected from the caudal vein with sterile plastic syringes. A portion of the blood was placed into K_3_EDTA tubes for hematological analyses, which included red blood cell (RBC) counts, hemoglobin (Hb) concentration, hematocrit (Ht), and erythrocyte indices [mean corpuscular volume (MCV), mean corpuscular hemoglobin (MCH), and mean corpuscular hemoglobin concentration (MCHC)]. Immunological assays performed from the same samples included white blood cell (WBC) counts, oxidative radical production assessed via the nitroblue tetrazolium (NBT) test, and evaluation of phagocytic activity (PA) and phagocytic index (PI). The remaining blood was transferred into plain serum tubes, allowed to clot at room temperature for 30 min, and centrifuged at 5000× *g* for 10 min. The resulting sera were stored at −20 °C until analysis of humoral immune parameters, including total protein (TP), immunoglobulin M (IgM), bactericidal activity (BA), lysozyme (LYZ), and myeloperoxidase (MPO).

Following blood sampling, the liver, head kidney, and spleen were excised, rinsed with physiological saline (0.9% NaCl), and stored at −40 °C until biochemical analyses were conducted within 1 month of collection. Tissue samples were homogenized in a Teflon–glass homogenizer using 1.15% KCl buffer at a 1:10 (*w*/*v*) dilution. The homogenates were then centrifuged at 18,000× *g* for 30 min at 4 °C. The supernatants were analyzed for malondialdehyde (MDA) concentration and antioxidant enzyme activities, including superoxide dismutase (SOD), catalase (CAT), and glutathione peroxidase (GSH-Px) [21].

Additionally, twenty fish per group were intraperitoneally challenged with *Aeromonas salmonicida* subsp. *achromogenes*. Mortality was monitored daily for 14 days, and cumulative mortality rates were calculated to assess disease resistance.

### 2.4. Growth Parameters

Growth performance parameters, such as weight gain (WG), specific growth rate (SGR), and feed conversion ratio (FCR), were determined using the following equations:

WG (g) = Wt − W0

SGR (%) = 100 × (ln Wt − ln W0)/t

FCR = FI/(Wt − W0)

where

Wt: Final average body weight (g);
W0: Initial mean body weight (g);
t: Experimental period (days);
FI: Feed intake (g).

### 2.5. Hematological Analysis

Red blood cell (RBC) counts were determined using a hemocytometer and Natt and Herrick diluting fluid. Hemoglobin (Hb) concentration was measured spectrophotometrically by assessing the absorbance of Drabkin’s reagent at 540 nm. Hematocrit (Ht) values were obtained through the microhematocrit centrifugation method. Erythrocyte indices, including mean corpuscular volume (MCV), mean corpuscular hemoglobin (MCH), and mean corpuscular hemoglobin concentration (MCHC), were calculated using established equations based on the measured values of Ht, RBC, and Hb [22].

MCV (µm^3^) = (Hematocrit × 10)/RBC count

MCH (pg) = (Hemoglobin × 10)/RBC count

MCHC (%) = (Hemoglobin × 100)/Hematocrit

### 2.6. Immunological Analysis

#### 2.6.1. WBC

White blood cell (WBC) counts were determined simultaneously with RBC counts using the Natt and Herrick method.

#### 2.6.2. Oxidative Radical Production of Neutrophil (NBT Assay)

The production of reactive oxygen species (ROS) by phagocytes was assessed using the nitroblue tetrazolium (NBT) reduction test. For this, 0.1 mL of fresh blood was added to each well of a microtiter plate, and an equal volume of 0.2% NBT solution was included. The mixture was incubated at room temperature for 30 min to allow the reaction to proceed. Then, 0.05 mL of the cell-NBT suspension was transferred into a glass tube containing 1.0 mL of N, N-dimethylformamide. After centrifugation, the absorbance of the supernatant was measured at 620 nm using a 1.0 mL cuvette [23].

#### 2.6.3. Phagocytic Activity (PA) and Phagocytic Index (PI)

Phagocytic activity (PA) and phagocytic index (PI) were assessed following the method of Siwicki and Anderson [24], using freshly collected heparinized blood. Briefly, 0.1 mL of blood was mixed with 0.1 mL of phosphate-buffered saline (PBS) containing bacterial cells at a concentration of 1 × 10^7^ cells. The mixtures were incubated in microplate wells for 30 min with gentle mixing. After incubation, 0.05 mL of the suspension was spread onto glass slides, air-dried, fixed in ethanol, and then stained with Giemsa solution for 10 min. Phagocytic cells and internalized bacteria were examined microscopically, and at least 100 phagocytes were evaluated per slide.

The following calculations were used:

PA (%) = (Number of phagocytic cells containing bacteria/Total number of phagocytes) × 100

PI = (Number of engulfed bacteria/Total number of phagocytic cells)

#### 2.6.4. Total Protein (TP) Level

The concentration of serum total protein (TP) was measured using a modified Biuret method. Briefly, 0.1 mL of serum was diluted with 0.9 mL of distilled water in a spectrophotometer tube and mixed gently by inversion. Subsequently, 4.0 mL of Biuret reagent was added, and the mixture was incubated in the dark at room temperature for 30 min. The absorbance of the resulting solution was then recorded at 540 nm using a spectrophotometer [23].

#### 2.6.5. Total Immunoglobulin (IgM) Level

The plasma immunoglobulin M (IgM) concentration was determined using the method of Siwicki et al. [23]. In this process, the total plasma protein content was first measured using a microprotein determination method (Biuret assay). IgM molecules were then selectively precipitated using a 12% (*w*/*v*) solution of polyethyleneglycol (Sigma, Darmstadt, Germany). The decrease in protein concentration after precipitation was taken as the IgM content.

#### 2.6.6. Serum Bactericidal Activity (BA)

Bactericidal activity (BA) of the serum was evaluated following the method of Kajita et al. [25], with minor modifications. Equal volumes (100 μL each) of serum and bacterial suspension were mixed thoroughly and incubated at 25 °C for 1 h. A blank control was prepared in parallel by replacing serum with sterile phosphate-buffered saline (PBS). After incubation, the mixtures were diluted tenfold with sterile PBS, and 100 μL aliquots of the serum–bacteria suspension were spread onto tryptic soy agar (TSA) plates. The plates were incubated at 25 °C for 24 h, after which the number of bacterial colonies was counted to determine the surviving viable bacteria.

#### 2.6.7. Serum Lysozyme (LYZ) Activity

Serum lysozyme activity was measured as described by Ellis [26]. Briefly, 200 μL of *Micrococcus lysodeikticus* (ATCC 4698) suspension (2 mg/mL) prepared in 0.05 M sodium phosphate buffer (pH 6.2) was dispensed into the wells of a microplate. To this, 50 μL of serum sample was added, and the reaction mixture was incubated at 25 °C. The absorbance was recorded at 450 nm after 0.5 and 10 min. One unit of lysozyme activity was defined as the amount of enzyme that causes a decrease in absorbance of 0.001 per minute per mL of serum.

#### 2.6.8. Serum Myeloperoxidase (MPO) Activity

Myeloperoxidase (MPO) activity was measured according to the method of Quade and Roth [27], with slight modifications as described by Sahoo et al. [28]. Briefly, 20 μL of serum was diluted in Hank’s Balanced Salt Solution (HBSS; Sigma) without Ca^2+^ and Mg^2+^ in 96-well microplates. Following this, 35 μL of 3,3′,5,5′-tetramethylbenzidine hydrochloride (TMB, 20 mM; Sigma) and hydrogen peroxide (H_2_O_2_, 5 mM) were added to initiate the reaction. After 2 min of incubation, the colorimetric reaction was terminated by adding 35 μL of 4 M sulfuric acid. The optical density was then measured at 450 nm using a microplate reader.

### 2.7. Challenge Test with Aeromonas salmonicida subsp. achromogenes

The median lethal dose (LD_50_) over seven days was first determined by intraperitoneal injection of serially diluted suspensions of *A. salmonicida* subsp. *achromogenes* into groups of ten fish. The bacterial strain (*A. salmonicida* subsp. *achromogenes* NCMB 1110) was cultured on blood agar at 22 °C, and freshly grown cells were suspended in sterile 0.85% sodium chloride (NaCl). The suspension was adjusted to an optical density of 0.9 at 610 nm (≈10^9^ cfu/mL) and then serially diluted to obtain concentrations ranging from 10^9^ to 10^4^ cfu/mL. Based on mortality observations, the 7-day LD_50_ was estimated as 3.12 × 10^6^ cfu/mL.

At the end of the feeding trial, thirty fish from each treatment group were intraperitoneally injected with 100 μL of phosphate-buffered saline (PBS) containing 3.12 × 10^6^ cfu/mL of live *A. salmonicida* subsp. *achromogenes*. After infection, the fish were kept on the basal control diet and observed daily for 14 days. The cause of death in moribund or dead fish was confirmed by re-isolation of the bacterium from liver and spleen tissues using standard microbiological methods.

Survival was calculated according to the formula described by Iswarya et al. [29]:

Survival (%) = (Number of surviving fish after challenge/Number of fish injected with *A. salmonicida* subsp. *achromogenes*) ×100.

### 2.8. Oxidative Stress and Antioxidant Capacity

#### 2.8.1. Tissue Malondialdehyde (MDA) Level

Lipid peroxidation in tissues was assessed by measuring malondialdehyde (MDA) using the thiobarbituric acid (TBA) assay, as described by Placer et al. [30]. The concentration of TBA-reactive substances was quantified spectrophotometrically by comparing absorbance values against a calibration curve prepared using malondialdehyde equivalents derived from the acid-catalyzed hydrolysis of 1,1,3,3-tetramethoxypropane.

#### 2.8.2. Tissue Superoxide Dismutase (SOD) Activity

SOD activity was assessed as described by Sun et al. [31]. The assay measures SOD’s ability to inhibit the reduction in nitroblue tetrazolium (NBT) by superoxide radicals produced in the xanthine–xanthine oxidase system. The reaction mixture included 0.1 mM xanthine, 0.1 mM EDTA, 50 mg/L bovine serum albumin, and 25 μM NBT. A total of 600 μL of this mixture was combined with 125 μL of sample supernatant or SOD standard, and the reaction was started by adding 25 μL of 9.9 nM xanthine oxidase solution. After incubation for 20 min at 25 °C, the reaction was stopped by adding 0.5 mL of 0.8 mM CuCl_2_. The absorbance was measured at 560 nm to determine SOD activity.

#### 2.8.3. Tissue Catalase (CAT) Activity

Catalase (CAT) activity was measured following the method of Aebi [32]. The principle involves monitoring the rate of hydrogen peroxide (H_2_O_2_) decomposition. For the assay, 2 mL of the sample was mixed with 1 mL of 40 mM H_2_O_2_ prepared in phosphate buffer (50 mM, pH 7.0). The decrease in absorbance at 240 nm was recorded for 3 min using a spectrophotometer, and enzyme activity was expressed as the rate constant of H_2_O_2_ decomposition. The results were expressed as k/mg protein, where k is the first-order rate constant.

#### 2.8.4. Tissue Glutathione Peroxidase (GSH-Px) Activity

Glutathione peroxidase (GSH-Px) activity was measured following Beutler [33]. The assay tracked NADPH oxidation at 340 nm. The reaction mixture included 50 mM potassium phosphate buffer (pH 7.0), 1 mM EDTA, 1 mM sodium azide, 0.2 mM NADPH, 1 mM GSH, 0.25 mM H_2_O_2_, and glutathione reductase at 1 EU/mL. A 0.1 mL enzyme sample was added to 0.8 mL of the reaction mixture, which was incubated for 5 min at 25 °C, with the reaction starting upon addition of 0.1 mL of peroxide. The decline in absorbance at 340 nm was recorded over 5 min, and activity was expressed as micromoles of NADPH oxidized per minute.

#### 2.8.5. Tissue Protein Content

The total protein concentration in tissue homogenates was measured using the Lowry method [34], with bovine serum albumin used as the standard.

### 2.9. Molecular Docking Studies

The chemical structure of EA, used as a ligand, was generated from SMILES data obtained from PubChem (https://pubchem.ncbi.nlm.nih.gov/, accessed on 20 June 2026), and energy minimization was performed using the ChemOffice 2022 program. PDB files of macromolecules were obtained from the Protein Data Bank (https://www.rcsb.org/), and Maestro Release 2026-1 (version 14.7) was modified and optimized as described in [35]. The grid box is positioned with 60 × 60 × 60 Å^3^ dimensions and 0.375 Å spacing, in accordance with the previously determined or predicted regions for the active site of the SOD (PDB ID: 3LSU), CAT (PDB ID: 1DGB), and GSH-Px (PDB ID: 2F8A), if any [36]. 50 runs were performed for each macromolecule using standard settings [37]. The Lamarckian Genetic Algorithm was preferred in all AutoDock studies, and both AutoDock (version 1.5.7) and Vina (version 1.1.2) results are presented in detail in the Section 3 [38,39]. For validation, cocrystal ligands were redocked onto target sites, and RMSD values were determined to be between 1.33 and 1.81 [40].

### 2.10. Statistical Analysis

The experiment was conducted using a 2 × 3 factorial design, including two stocking densities (SD; 25 and 100 kg/m^3^) and three dietary levels of EA (EA; 0, 50, and 100 mg/kg). The normality of the data was assessed using the Shapiro–Wilk test. To evaluate the main effects of SD and EA, as well as their interaction, a two-way analysis of variance (Two-Way ANOVA) was performed using the General Linear Model (GLM) procedure. Polynomial contrasts were applied to examine linear and quadratic trends associated with increasing EA levels. When significant interactions were detected, group comparisons were conducted using Tukey’s post hoc test. The statistical model used was as follows:

Y(ijk) = μ + SD_i_ + EA_j_ + (SD × EA)_ij_ + e(ijk),

where Y represents the response variable, μ is the overall mean, SD is the main effect of stocking density, EA is the main effect of ellagic acid supplementation, (SD × EA) is the interaction effect, and e is the residual error. To further validate differences among treatment groups, one-way ANOVA, followed by Tukey’s post hoc test, was applied to normally distributed data, whereas non-normally distributed data were analyzed using the Kruskal–Wallis test, followed by Bonferroni-adjusted multiple comparisons. Statistical significance was accepted at *p* < 0.05. The LD_50_ value for bacteria was calculated using Finney’s probit analysis [41].

## 3. Results

### 3.1. Growth Performance

**Figure 1:**
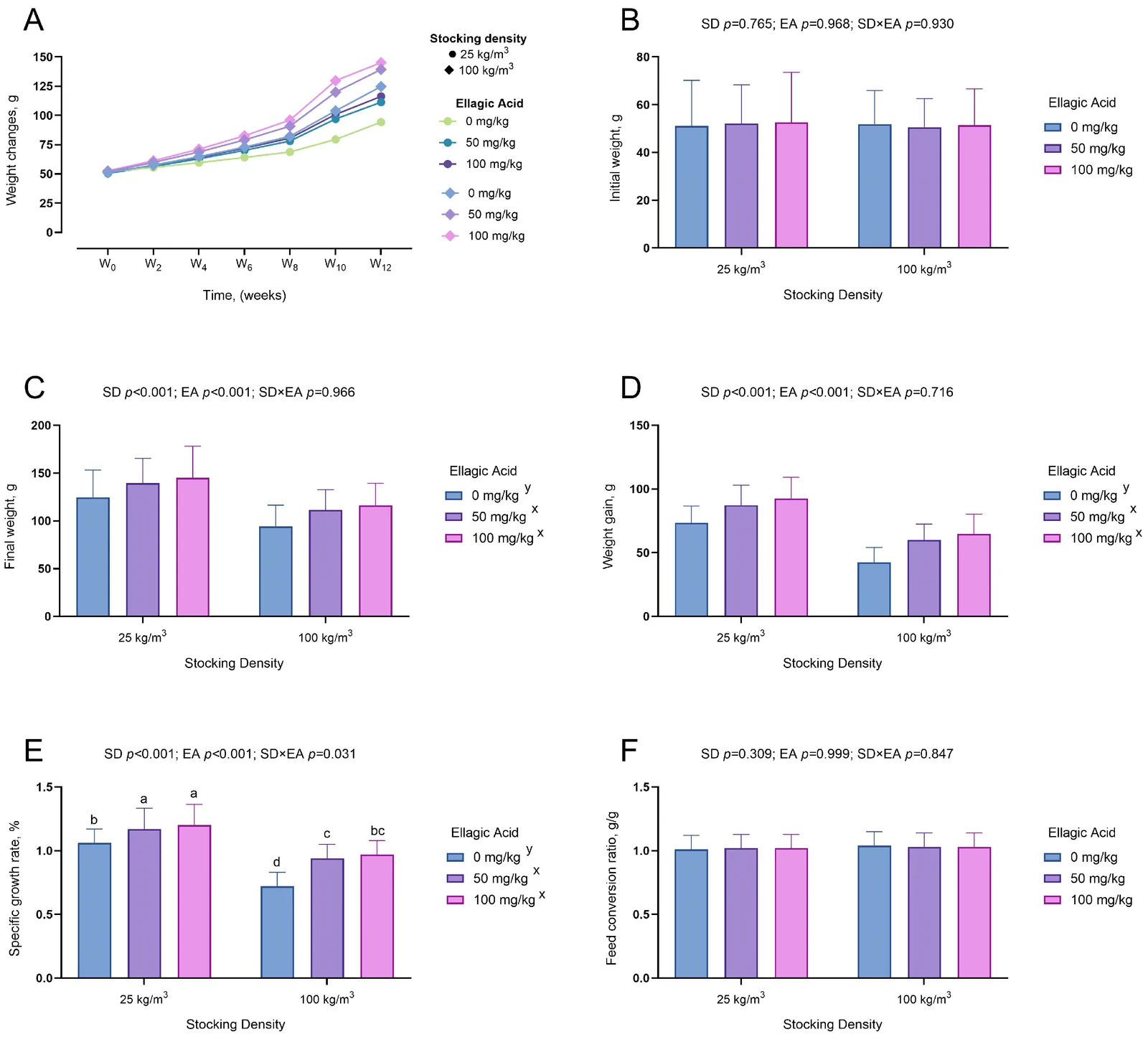
Effects of dietary ellagic acid (EA; 0, 50, or 100 mg/kg) supplementation on growth performance parameters [weight changes (**A**), initial weight (**B**), final weight (**C**), weight gain (**D**), SGR (**E**), and FCR (**F**)] in carp reared under different stocking densities (SD; 25 kg/m^3^ and 100 kg/m^3^). Values are presented as mean ± standard deviation. The *p*-values for the main effects of SD and EA, and the interaction between SD and EA (SD × EA), are presented. Different lowercase superscript letters (a–d) indicate significant differences among treatment combinations when a significant interaction (SD × EA) was detected, whereas different uppercase superscript letters (x, y, z) denote significant differences among EA doses.

Growth performance parameters are presented in Figure 1. Two-way ANOVA revealed that stocking density (SD) and dietary ellagic acid (EA) supplementation significantly affected final weight, weight gain, and specific growth rate (SGR) (*p* < 0.05). Fish reared at high stocking density (100 kg/m^3^) exhibited significantly lower final weight, weight gain, and SGR than those maintained at low stocking density (25 kg/m^3^), indicating impaired growth performance under crowding stress. Dietary EA supplementation significantly improved weight gain and SGR (*p* < 0.05), with increasing EA levels yielding progressively greater improvements in these parameters. Polynomial contrast analysis confirmed significant linear effects of EA on weight gain and SGR (*p* < 0.05), whereas no consistent quadratic responses were detected. A significant SD × EA interaction was observed only for SGR, indicating that the response to dietary EA varied with SD. Under HSD, fish receiving 100 mg/kg EA exhibited the greatest improvement in SGR compared with the non-supplemented group. No significant effects of SD, dietary EA supplementation, or their interaction were detected for feed conversion ratio (FCR) (*p* > 0.05).

### 3.2. Hematological Parameters

The impact of SD, dietary EA, and their interaction on hematological variables is summarized in Table 1. All measured hematological parameters were significantly affected at the HSD (*p* < 0.05). In contrast, fish raised in high-density conditions (50 and 100 kg/m^3^) exhibited lower RBC counts, Hb levels, Ht values, and MCV compared to low-density conditions (25 kg/m^3^); however, both MCH and MCHC were significantly higher under high-density conditions. All hematological indices responded significantly to dietary EA supplementation (*p* < 0.05). RBC, Hb, Ht, and MCV consistently increased with higher EA levels across all treatments, whereas MCH and MCHC decreased. The highest Ht and MCV values were recorded in the 100 mg/kg EA group, whereas the lowest MCHC was observed at the same supplementation level. A significant SD × EA interaction was present for Hb, MCV, and MCHC (*p* < 0.05), indicating that the response to EA depended on SD. At low SD, Hb remained relatively stable across EA treatments, but increased progressively with dietary EA at HSD. Similarly, MCV and MCHC showed a density-dependent response. In fish at 100 kg/m^3^, EA supplementation significantly increased MCV and decreased MCHC compared with the 0 mg/kg high-density group. Polynomial contrast analysis revealed a significant linear effect of EA on RBC (*p* = 0.013) at both stocking densities. At 25 kg/m^3^, higher EA levels led to linear increases in RBC and Ht, and linear reductions in MCH and MCHC. RBC, Hb, Ht, and MCV increased linearly at 100 kg/m^3^, while MCHC decreased linearly. No significant quadratic effects were found for any hematological parameter. In conclusion, the overall results of this study show that HSD negatively affected hematological status in carp blood, whereas dietary EA supplementation positively affected various erythrocyte-related parameters and partially alleviated the density-associated deterioration.

### 3.3. Immunological Parameters

The effects of SD, dietary EA, and their interaction on immunological parameters are presented in Table 2 and Table 3. All measured variables, including WBC, NBT, PA, PI, TP, IgM, BA, LYZ activity, and MPO activity, were significantly influenced by both SD and EA supplementation (*p* < 0.001). Fish reared at high stocking density (100 kg/m^3^) exhibited significantly lower values for all immunological parameters compared to those maintained at low density (25 kg/m^3^), indicating a marked suppression of immune function under crowding stress. In contrast, dietary EA supplementation significantly improved all immune-related variables in a dose-dependent manner, with the highest values generally observed in the 100 mg/kg EA group. Significant SD × EA interactions were detected for PA, PI, TP, BA, LYZ, and MPO (*p* < 0.05), demonstrating that the magnitude of EA effects depended on SD. In particular, EA supplementation resulted in more pronounced improvements at low SD, although clear beneficial effects were also observed at HSD. Despite these improvements, values in high-density groups generally remained lower than those in low-density groups. For WBC, NBT, and IgM, no significant interaction was observed (*p* > 0.05), indicating that the positive effects of EA on these parameters were consistent across stocking densities. Polynomial contrast analysis revealed significant linear increases in all immunological parameters with increasing EA levels at both stocking densities (*p* < 0.001), while no significant quadratic effects were detected. This indicates a steady, dose-dependent enhancement of immune responses with EA supplementation. Overall, these findings demonstrate that HSD induces a substantial immunosuppressive effect in common carp, whereas dietary EA effectively enhances both cellular and humoral immune responses, partially mitigating the adverse effects of crowding stress.

### 3.4. Challenge Test with Aeromonas salmonicida subsp. achromogenes

**Figure 2:**
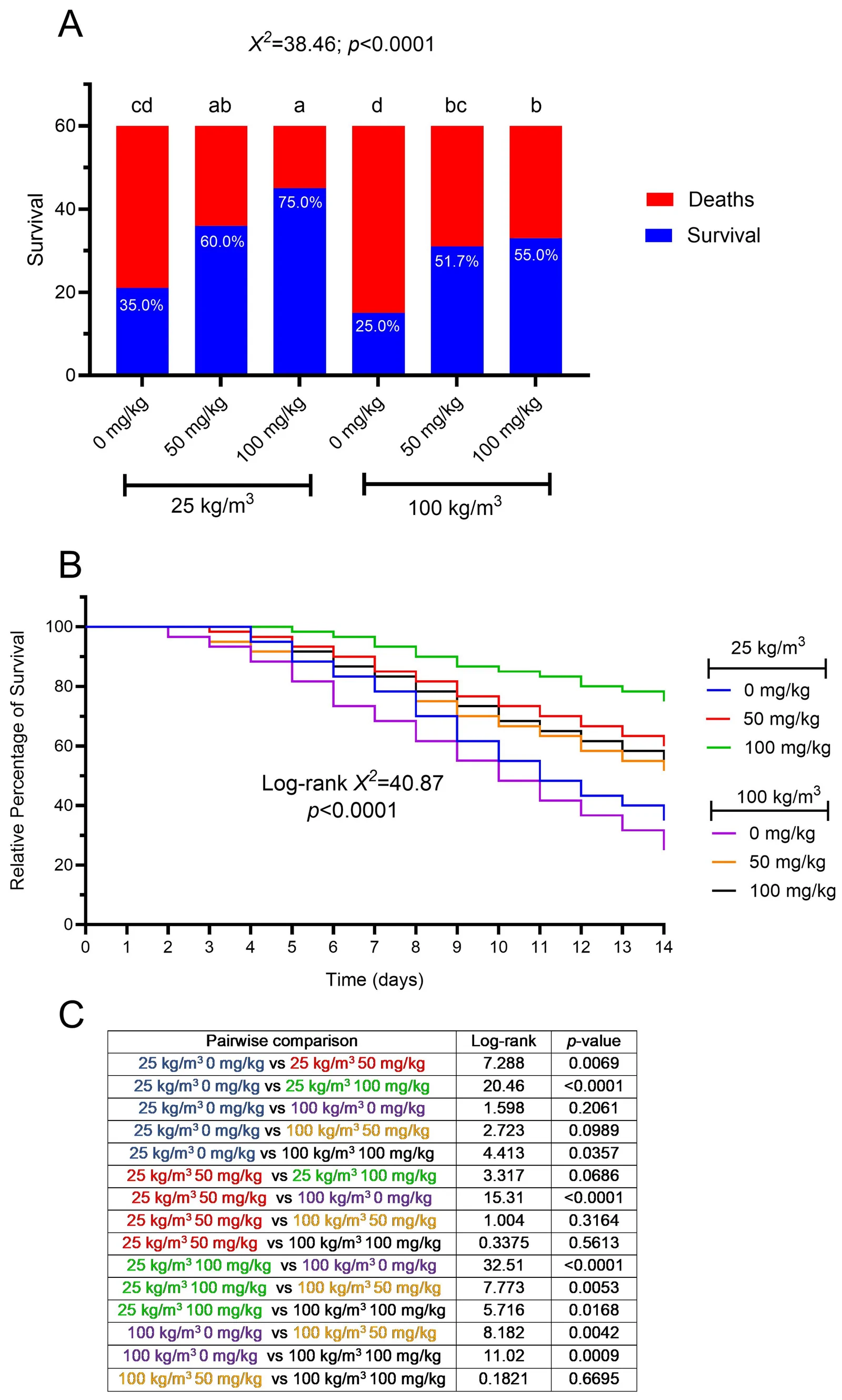
Effects of dietary ellagic acid (EA; 0, 50, or 100 mg/kg) supplementation on survival of carp reared under different stocking densities (SD; 25 and 100 kg/m^3^). (**A**) Survival and mortality percentages of fish in each experimental group. Differences in survival rates among groups were evaluated using the chi-square (χ^2^) test. Bars sharing different lowercase superscript letters (a–d) indicate statistically significant differences among treatment groups (*p* < 0.05). (**B**) Kaplan–Meier survival curves showing the cumulative survival probability over the experimental period. Differences in survival distributions among groups were assessed using the log-rank chi-square test. (**C**) Pairwise comparisons of survival curves among experimental groups using the log-rank chi-square test, with corresponding χ^2^ statistics and *p*-values presented.

Survival outcomes are presented in Figure 2. SD had a significant effect on survival, with fish reared at 100 kg/m^3^ exhibiting significantly lower survival than those at 25 kg/m^3^ (χ^2^ test, *p* < 0.05). Dietary EA supplementation significantly improved survival across both stocking densities, with the highest survival observed in the 100 mg/kg EA group. Kaplan–Meier survival analysis revealed clear separation among treatment groups, and log-rank test results confirmed significant differences in survival distributions (*p* < 0.05). Pairwise comparisons indicated that EA supplementation, particularly at 100 mg/kg, significantly enhanced survival under high-density conditions. These findings demonstrate that EA exerts a protective effect against density-induced mortality, with a clear dose-dependent pattern.

### 3.5. Oxidative Stress and Antioxidant Capacity

**Figure 3:**
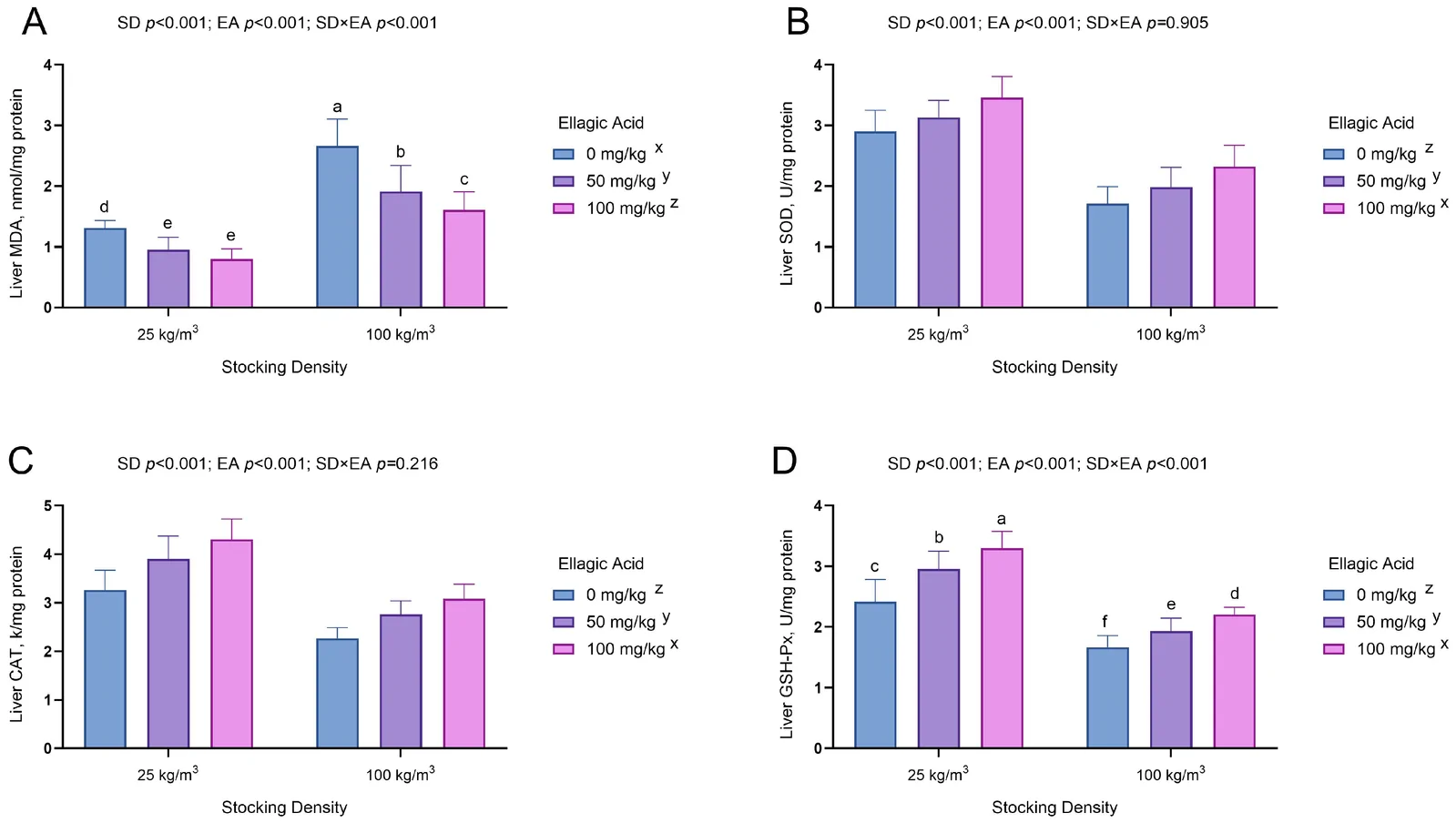
Effects of dietary ellagic acid (EA; 0, 50, or 100 mg/kg) supplementation on hepatic antioxidant parameters [Liver MDA (**A**), liver SOD (**B**), liver CAT (**C**), and liver GSH-Px (**D**) activities] in carp reared under different stocking densities (SD; 25 kg/m^3^ and 100 kg/m^3^). Values are presented as mean ± standard deviation. The *p*-values for the main effects of SD and EA, and the interaction between SD and EA (SD × EA), are presented. Different lowercase superscript letters (a–f) indicate significant differences among treatment combinations when a significant interaction (SD × EA) was detected, whereas different uppercase superscript letters (x, y, z) denote significant differences among EA doses.

Figure 3 shows liver MDA levels and antioxidant enzyme activities (SOD, CAT, and GSH-Px) across different experimental groups. Hepatic oxidative stress parameters were significantly influenced by SD, EA supplementation, and their interaction (*p* < 0.05). Fish exposed to HSD had significantly higher MDA levels and lower SOD, CAT, and GSH-Px activities compared to the low-density group, indicating increased oxidative stress. Dietary EA supplementation significantly decreased MDA levels while enhancing antioxidant enzyme activities in a dose-dependent manner. Polynomial contrast analysis revealed significant linear decreases in MDA levels and linear increases in SOD, CAT, and GSH-Px activities with increasing EA levels (*p* < 0.05). A significant SD × EA interaction indicated that EA’s antioxidative effects were more pronounced under high-density conditions. Despite these improvements, antioxidant parameters in high-density groups did not fully reach the levels observed at low density.

**Figure 4:**
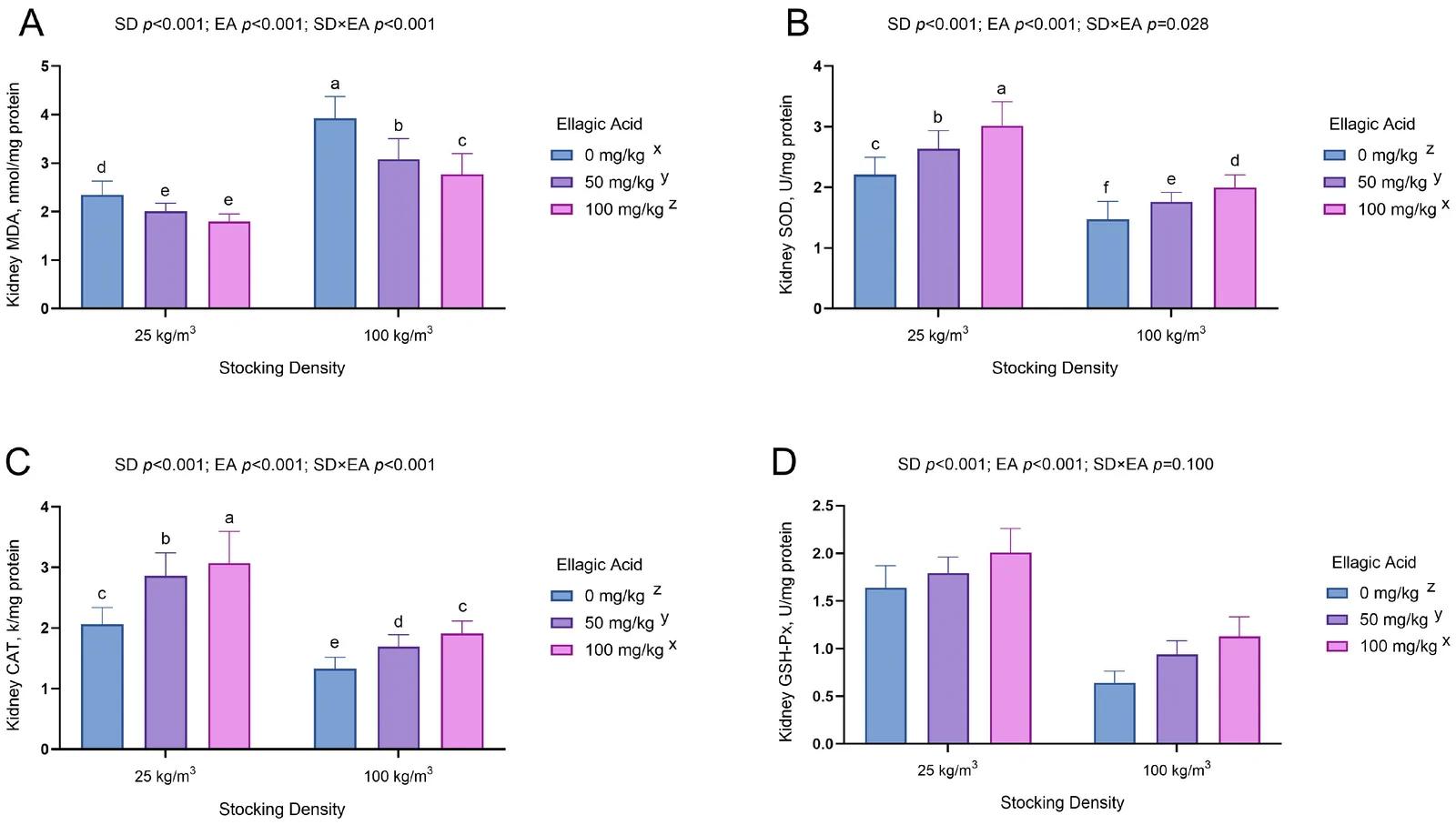
Effects of dietary ellagic acid (EA; 0, 50, or 100 mg/kg) supplementation on head kidney antioxidant parameters [kidney MDA (**A**), kidney SOD (**B**), kidney CAT (**C**), and kidney GSH-Px (**D**) activities] in carp reared under different stocking densities (SD; 25 kg/m^3^ and 100 kg/m^3^). Values are presented as mean ± standard deviation. The *p*-values for the main effects of SD and EA, and the interaction between SD and EA (SD × EA), are presented. Different lowercase superscript letters (a–f) indicate significant differences among treatment combinations when a significant interaction (SD × EA) was detected, whereas different uppercase superscript letters (x, y, z) denote significant differences among EA doses.

Figure 4 shows oxidative stress markers in head kidney tissue, including MDA levels and antioxidant enzyme activities. In this tissue, both SD and EA had significant main effects on oxidative stress markers (*p* < 0.05). HSD increased MDA levels and decreased antioxidant enzyme activities, confirming a tissue-specific oxidative imbalance under stress conditions. EA supplementation significantly improved antioxidant status, as shown by reduced MDA levels and increased activities of SOD, CAT, and GSH-Px. Linear trend analysis confirmed dose-dependent improvements (*p* < 0.05). The SD × EA interaction was significant, indicating that EA was more effective at reducing oxidative stress in fish under high-density conditions. However, complete normalization to control levels was not achieved.

**Figure 5:**
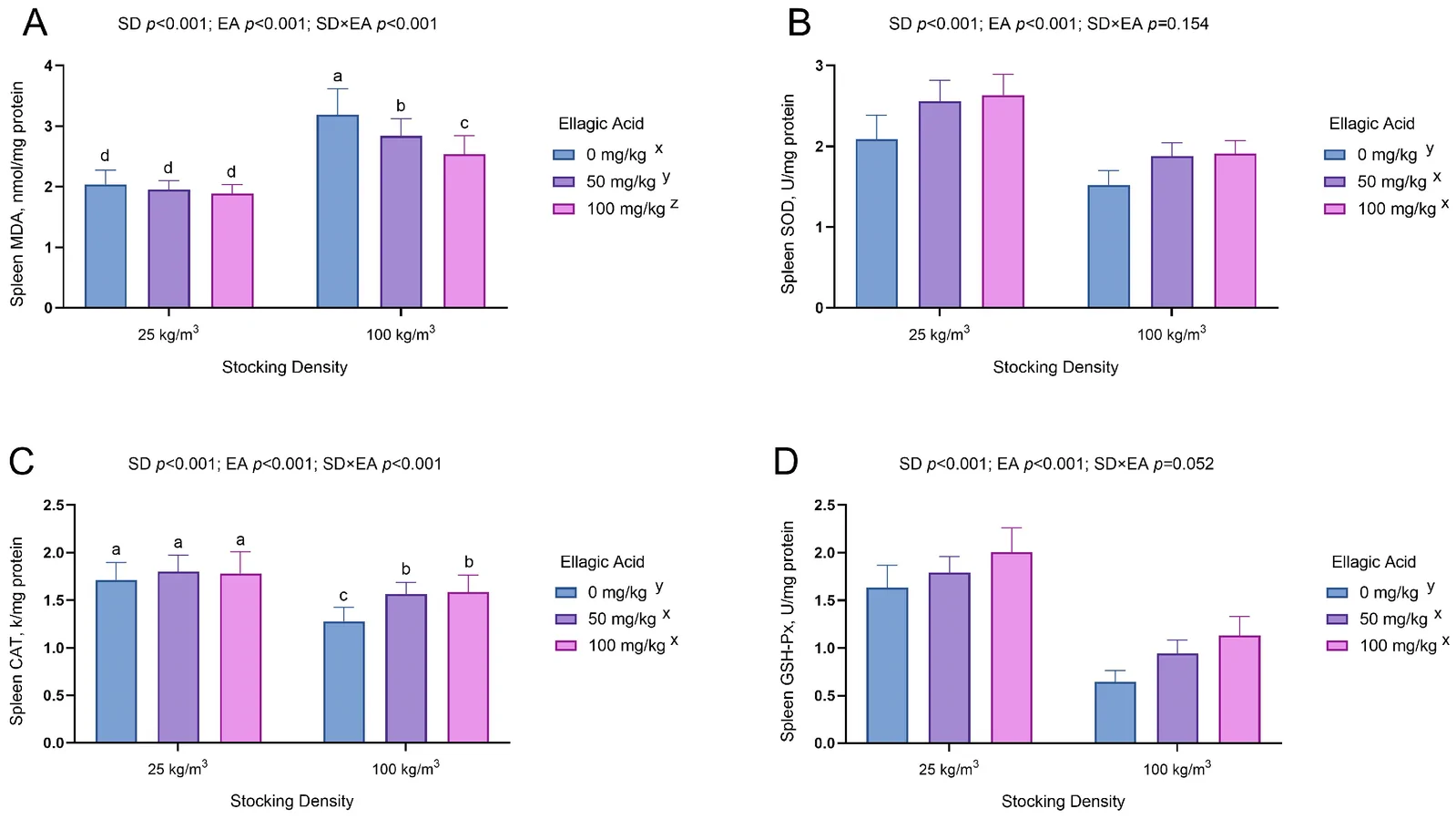
Effects of dietary ellagic acid (EA; 0, 50, or 100 mg/kg) supplementation on spleen antioxidant parameters [spleen MDA (**A**), spleen SOD (**B**), spleen CAT (**C**), and spleen GSH-Px (**D**) activities] in carp reared under different stocking densities (SD; 25 kg/m^3^ and 100 kg/m^3^). Values are presented as mean ± standard deviation. The *p*-values for the main effects of SD and EA, and the interaction between SD and EA (SD × EA), are presented Different lowercase superscript letters (a–d) indicate significant differences among treatment combinations when a significant interaction (SD × EA) was detected, whereas different uppercase superscript letters (x, y, z) denote significant differences among EA doses.

Figure 5 shows MDA levels in the spleen and antioxidant enzyme activities across different treatments. Spleen antioxidant responses were similar to those seen in liver and head kidney tissues. HSD significantly increased MDA levels and decreased antioxidant enzyme activities (*p* < 0.05), indicating systemic oxidative stress. EA supplementation significantly reduced MDA levels and increased SOD, CAT, and GSH-Px activities in a dose-dependent way. All parameters showed significant linear responses (*p* < 0.05), and no consistent quadratic effects were found. The SD × EA interaction also showed that EA supplementation was especially effective under high-density conditions, although antioxidant parameters remained only partially lower than in low-density groups.

**Figure 6:**
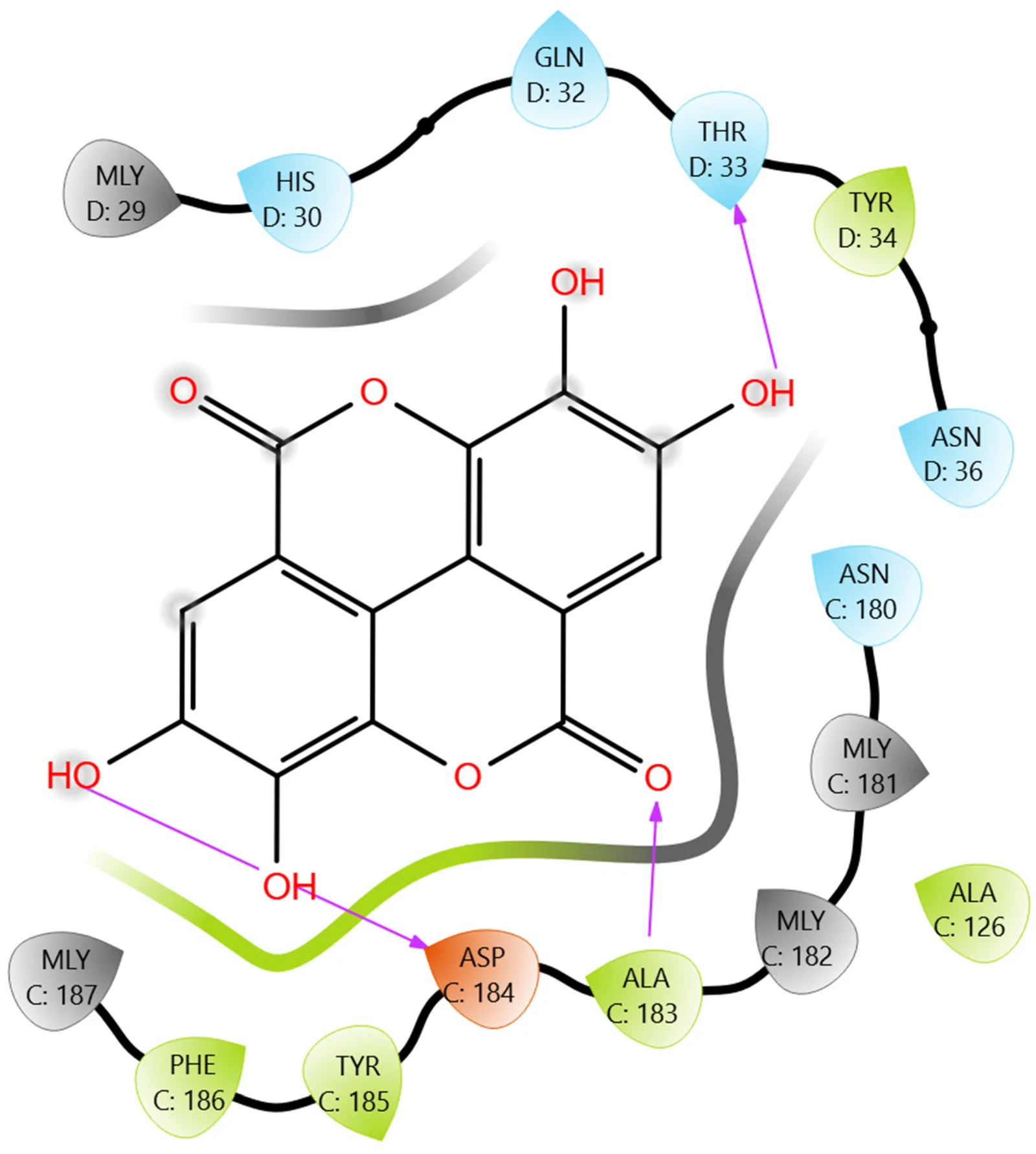
2D interaction diagram with SOD for EA.

**Figure 7:**
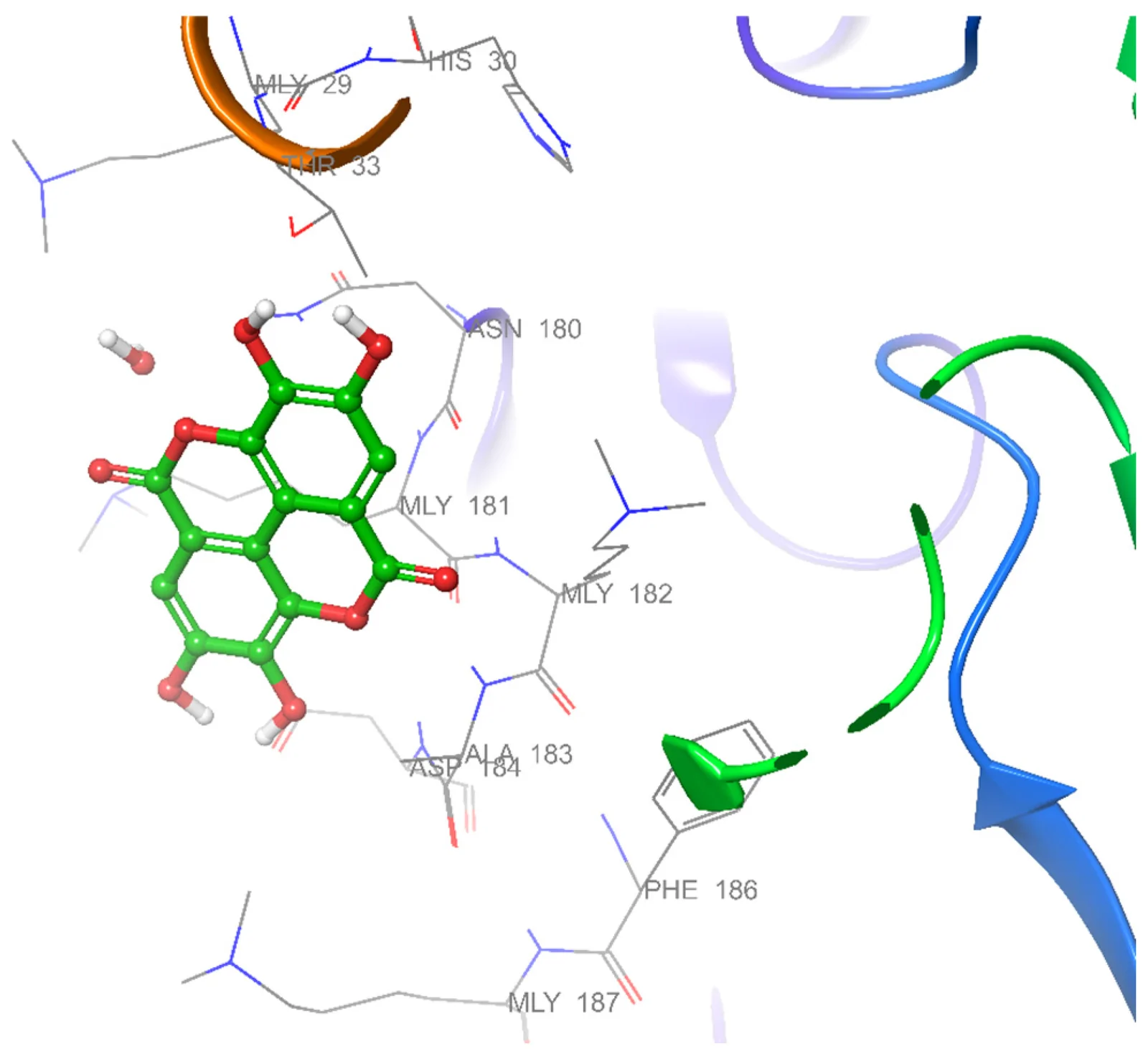
3D interaction diagram with SOD for EA.

**Figure 8:**
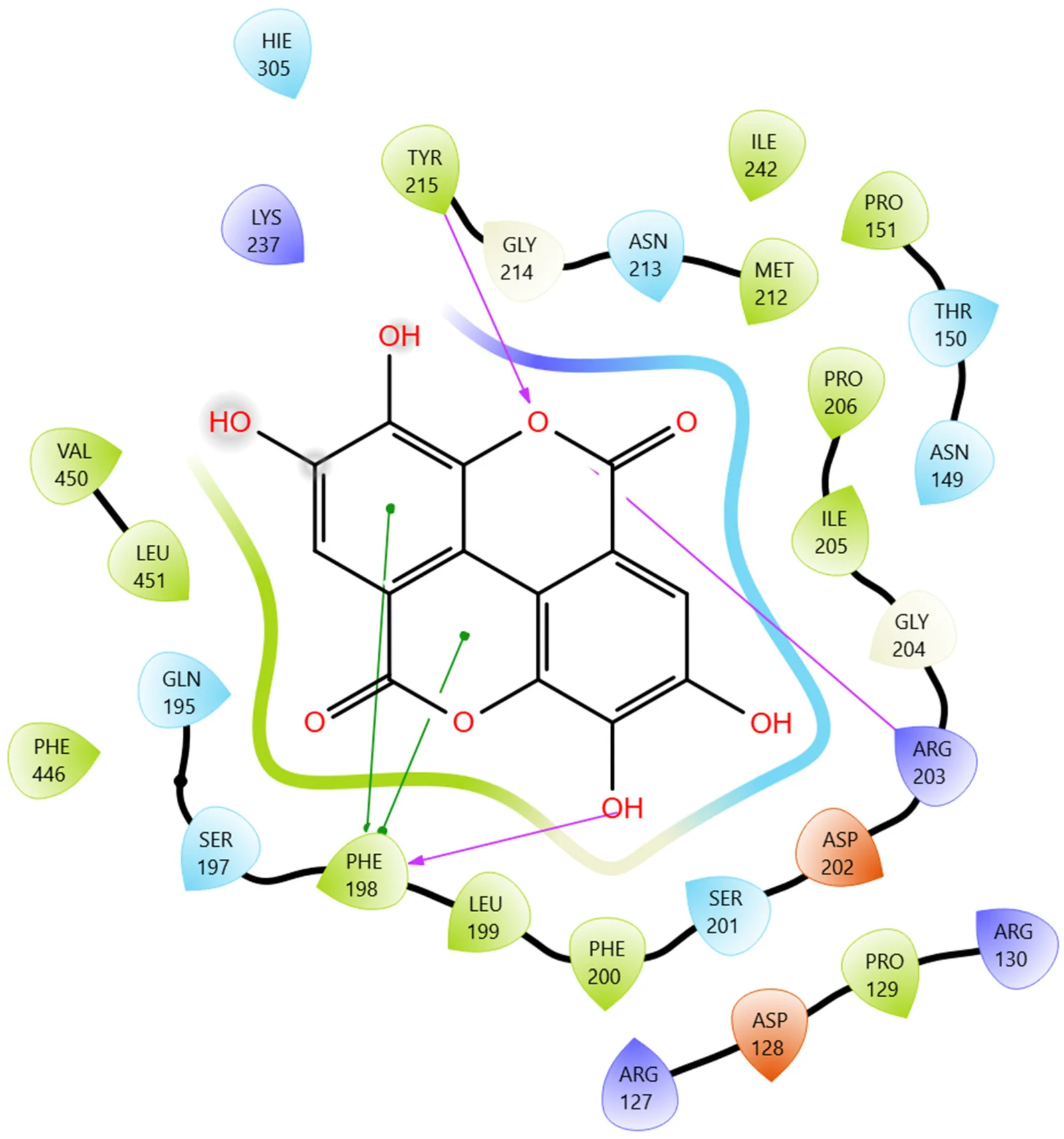
2D interaction diagram with CAT for EA.

**Figure 9:**
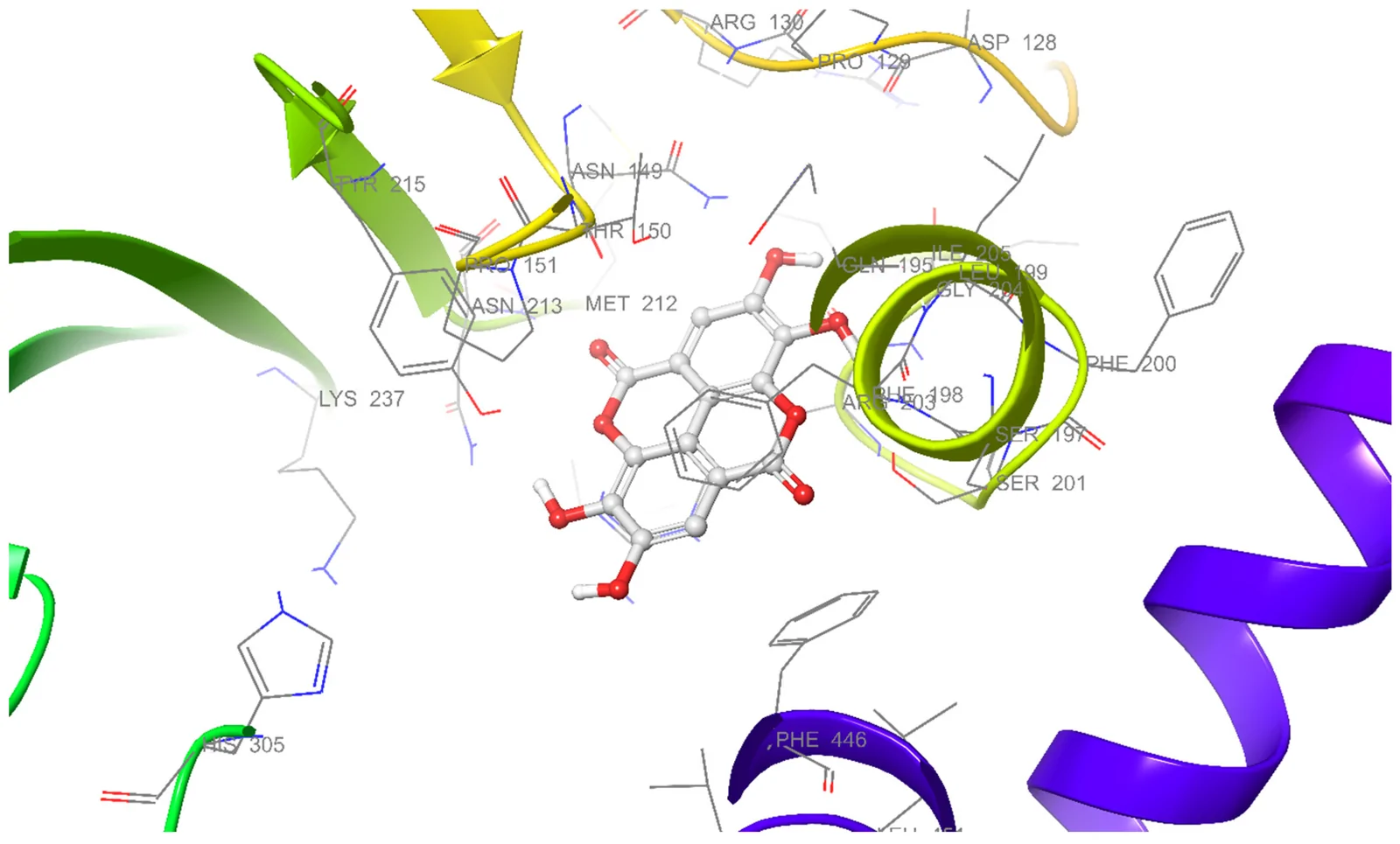
3D interaction diagram with CAT for EA.

**Figure 10:**
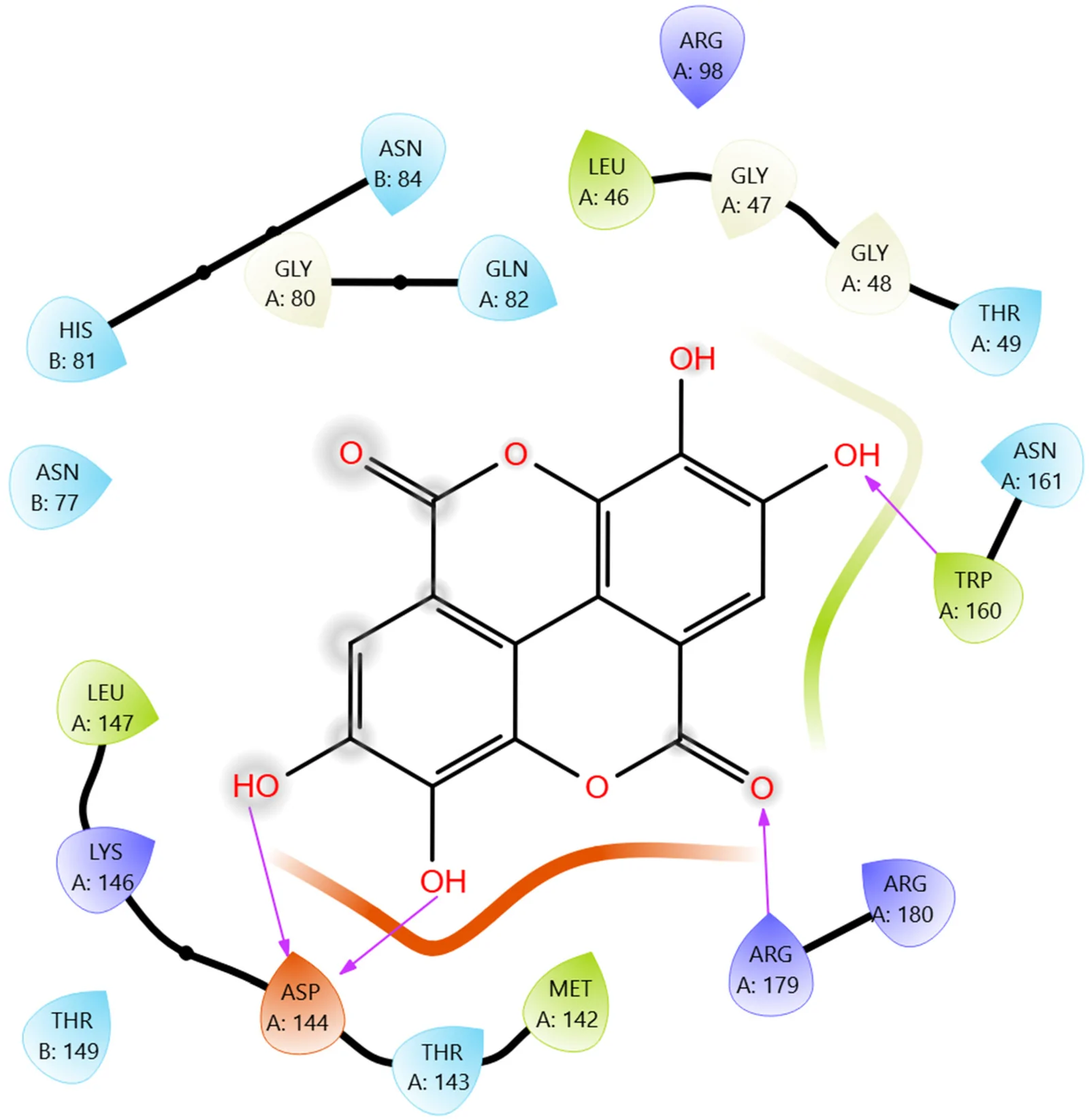
2D interaction diagram with GSH-Px for EA.

**Figure 11:**
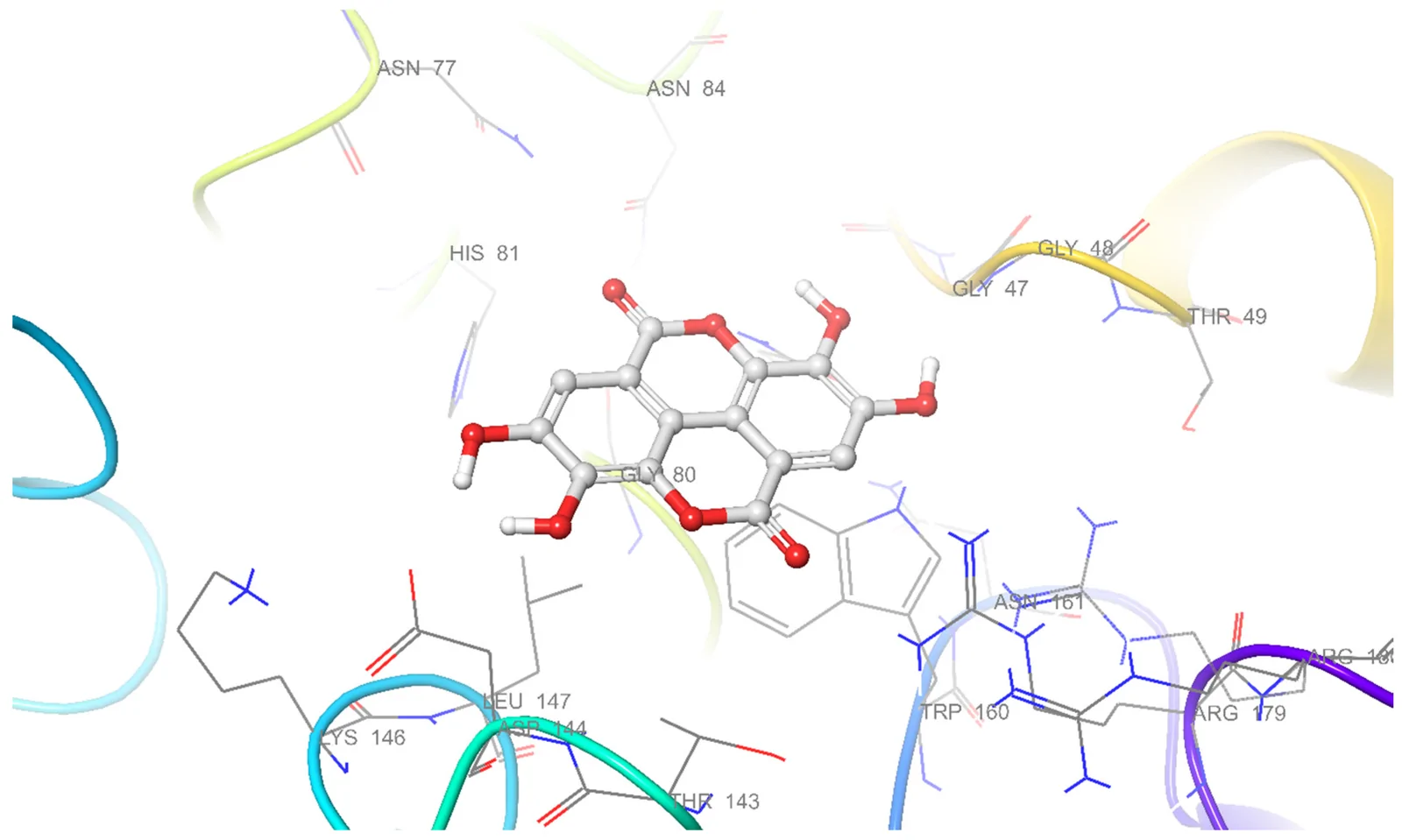
3D interaction diagram with GSH-Px for EA.

It has been determined that EA forms hydrogen bonds with the following residues of SOD: ALA183, C: ASP184, D: THR33; with the following residues of CAT: PHE198, ARG203, TYR215; and with the following residues of GSH-Px: ASP144, A: TRP160, A: ARG179. Additionally, it has been found that EA interacts with the PHE198 residue of CAT via a pi-pi interaction. It has been calculated that EA will exhibit micromolar-level activity on all these macromolecules (Table 4 and Figure 6, Figure 7, Figure 8, Figure 9, Figure 10 and Figure 11).

## 4. Discussion

Growth performance is widely recognized as a sensitive indicator of long-term husbandry stress in aquaculture systems. In this study, HSD significantly decreased growth-related parameters and feed efficiency in common carp. Similar density-dependent growth impairments have been consistently documented in previous research, in which crowding stress increased metabolic demands and reduced energy allocation to somatic growth with prolonged exposure [1,42]. Elevated SD has also been shown to disrupt feeding behavior and nutrient absorption, thereby negatively impacting growth performance under intensive rearing conditions. Recent studies in common carp have likewise demonstrated that intensive stocking density impairs growth performance, disrupts antioxidant status, and alters intestinal microbial communities, further confirming the detrimental physiological effects of chronic crowding stress under intensive aquaculture conditions [4]. These findings indicate that chronic husbandry stress extends beyond oxidative imbalance, adversely affecting growth, hematological status, immune competence, and disease resistance by disrupting multiple physiological systems. Dietary EA partially alleviated these adverse effects, suggesting that its protective action involves maintaining overall physiological homeostasis rather than merely scavenging reactive oxygen species. Collectively, these findings support the use of nutritional strategies targeting redox homeostasis to enhance immune function, disease resistance, and physiological resilience in intensive aquaculture systems.

EA supplementation clearly improved growth performance, especially under HSD. Although direct studies on the role of EA in fish growth are limited, many investigations have shown that plant-derived antioxidants and polyphenolic compounds can counteract stress-related growth suppression. These compounds are known to reduce oxidative damage and improve metabolic efficiency, indirectly promoting growth and feed utilization in challenging rearing conditions [11]. Supporting this idea, dietary curcumin supplementation has been shown to increase body weight gain and feed intake in rainbow trout raised under HSD, primarily by enhancing antioxidant defenses and improving physiological resilience to crowding stress [43]. Given the strong antioxidant properties of EA, the growth-promoting effects observed in this study are likely mediated by similar protective mechanisms. Overall, these findings suggest that EA supplementation can help reduce growth depression caused by high density by supporting metabolic balance and enhancing the fish’s ability to cope with chronic husbandry stress.

In this study, HSD caused significant alterations in hematological indices, reflecting stress-induced disturbances in oxygen transport capacity and immune-related blood components. Changes in erythrocyte and leukocyte profiles are well-established physiological responses to chronic stress in fish and have been widely reported under intensive rearing conditions [1,2]. Dietary EA supplementation partially restored these hematological alterations, particularly under HSD, indicating improved physiological adaptation to crowding stress. Previous studies have demonstrated that plant-derived antioxidants and polyphenolic compounds protect blood cells against oxidative damage and support hematopoietic function in fish exposed to environmental and husbandry-related stressors [11,44]. Collectively, these findings suggest that the beneficial effects of EA extend beyond antioxidant activity, contributing to the preservation of hematological homeostasis and overall physiological resilience under intensive culture conditions.

HSD is a well-known chronic stressor that can influence innate immune function in fish. In this study, immune-related parameters were suppressed during HSD, consistent with evidence that prolonged stress responses, primarily mediated by corticosteroids, can reduce disease resistance and alter key humoral and cellular immune markers [2]. Additionally, studies on experimental crowding have shown measurable changes in nonspecific defense markers such as lysozyme and complement activity under sustained high-density conditions, supporting the idea that crowding can impair innate immune competence [45].

Dietary EA supplementation enhanced immune profiles, especially under HSD. This pattern aligns with broader research showing that plant-derived compounds can boost nonspecific immune responses and support host resistance in fish, often reflected in increases in traditional innate immune markers (e.g., LYZ activity, respiratory burst, and PA) [11,46,47,48]. Overall, our findings indicate that EA may help mitigate density-related immunosuppression, likely by supporting redox balance and maintaining immune cell function during chronic husbandry stress.

Survival is commonly used as an indicator of host resistance, reflecting the combined effects of immune capacity, physiological resilience, and stress tolerance in fish. In this study, HSD significantly reduced survival values, suggesting greater vulnerability to stress-related mortality under crowded rearing conditions. Similar declines in survival and disease resistance at high stocking densities have been noted in previous research, in which chronic stress exposure impaired the immune response and increased susceptibility to pathogens [1,45]. The improved resistance to *Aeromonas salmonicida* subsp. *achromogenes* challenge further suggests that the benefits of EA extend beyond restoring biochemical biomarkers. The simultaneous improvement in innate immune parameters and pathogen resistance indicates that preserving redox homeostasis may directly contribute to enhanced functional immunity during chronic husbandry stress.

Dietary EA supplementation significantly increased survival, with the greatest improvement seen in fish reared at high stocking densities. Similar improvements in survival have been reported in salmonids under crowding stress, where dietary antioxidants effectively reduced high-density-related mortality. In rainbow trout, antioxidant-enriched diets were shown to enhance stress tolerance by improving immune responses and reducing oxidative damage, thereby supporting overall physiological stability during intensive rearing. The higher survival values observed in EA-supplemented carp in this study align with these findings and indicate a combined enhancement of immune function and stress resilience. Additionally, previous studies have demonstrated that plant-derived antioxidants and immunostimulatory compounds can enhance fish survival by strengthening innate immune defenses and maintaining redox balance during prolonged stress [11,47]. Collectively, these results suggest that EA supplementation enhances the host’s defense mechanisms and supports physiological homeostasis under chronic husbandry stress, thereby improving survival in densely stocked fish.

Oxidative stress is a common physiological consequence of chronic husbandry-related stressors, such as crowding, which promote excessive production of reactive oxygen species (ROS) and place high demands on endogenous antioxidant defense systems. In the present study, exposure to HSD significantly reduced antioxidant capacity, as evidenced by decreased activities of key antioxidant enzymes and a shift toward a more oxidative internal state. These findings indicate that prolonged stress overwhelms both enzymatic and non-enzymatic antioxidant defenses, disrupts redox homeostasis, and increases the risk of cellular damage, supporting the widely accepted concept that sustained oxidative pressure compromises cellular integrity and physiological stability in aquatic organisms [44].

Findings from intensive aquaculture research consistently demonstrate that increased stocking density suppresses antioxidant defenses and enhances oxidative stress in fish. Similarly, Rong et al. [10] demonstrated that elevated stocking density significantly altered antioxidant status and lipid metabolism in common carp, confirming that chronic crowding stress disrupts redox homeostasis in this species. Long-term crowding reduces the activities of major antioxidant enzymes, including superoxide dismutase, catalase, and glutathione, while increasing physiological stress responses and lipid peroxidation, thereby indicating a marked oxidative imbalance [42]. Similar responses have been reported in rainbow trout, where HSD increased lipid peroxidation and reduced antioxidant enzyme activities. Dietary supplementation with polyphenolic antioxidants such as curcumin [43] and lycopene [49] has been shown to decrease MDA levels and restore antioxidant enzyme activities, mainly through activation of redox-sensitive pathways, particularly the Nrf2/HO-1 signaling pathway, in addition to their direct ROS-scavenging capacity [43,49]. These findings collectively support the use of nutritional antioxidants to mitigate density-induced oxidative stress by reinforcing endogenous antioxidant defense mechanisms.

Dietary EA supplementation significantly improved antioxidant capacity in the present study, with the greatest protective effects observed under HSD conditions. Plant-derived polyphenols are widely recognized for their ability to neutralize reactive oxygen species and support endogenous antioxidant defenses [11]. Their protective efficacy generally becomes more evident as oxidative stress intensifies, which explains why the beneficial effects of EA were more pronounced at high stocking density than at low density. Overall, the present findings demonstrate that HSD adversely affects growth, hematological health, immune function, antioxidant capacity, and survival in common carp (*Cyprinus carpio*), whereas dietary EA supplementation effectively alleviates these detrimental effects by improving growth performance, stabilizing hematological parameters, enhancing immune responses, strengthening antioxidant defenses, and increasing physiological resilience, particularly under intensive rearing conditions. These findings further support the potential of EA as a functional feed additive for reducing density-induced oxidative stress in common carp culture.

Notably, the interaction between SD and dietary EA supplementation shows that EA’s beneficial effects are stronger at high stocking densities, highlighting its potential as a functional feed additive in intensive aquaculture systems. While fish reared at low stocking densities showed relatively stable physiological responses, EA supplementation offered additional support against stress-related disturbances.

The crystal structure of SOD with its ligand Glycerol (PDB: GOL) has been revealed, and the interaction domain is clearly stated. In previous studies on TYR178, MLY181, ASP184, and Na209, these residues have been emphasized as important for interactions (https://www.ebi.ac.uk/pdbe/entry/pdb/3lsu?activeTab=ligands&id=GOL, accessed on 20 June 2026). In this study, we determined that our ligand EA interacts with these regions of SOD in a similar manner. It has also been observed that it makes an H-bond in the same way as ASP184. EA exhibited favorable binding affinity toward SOD in the micromolar range.

The crystal structure of CAT with its ligand NADPH Dihydronicotinamide-adenine dinucleotide phosphate (PDB: NDP) has been revealed, and the interaction domain is clearly stated. In previous studies, HIS194, PHE198, SER201, ARG203, ASN213, LYS237, TRP303, PRO304, HIS305, GLN442, PHE446, and VAL450 have been emphasized as important for interaction (https://www.ebi.ac.uk/pdbe/entry/pdb/1dgb?activeTab=ligands&id=NDP, accessed on 20 June 2026). In this study, we determined that our ligand, EA, interacts with these CAT regions in a similar manner. It has also been observed to form an H-bond in the same way as PHE198 and ARG203. EA exhibited favorable binding affinity toward CAT in the micromolar range.

The crystal structure of GSH-Px with its ligand, malonic acid (PDB: MLA501), has been determined, and the interaction domain is clearly defined. In previous studies, MET142, THR143, ASP144, LEU147, and ARG179 have been emphasized as important for interactions (https://www.ebi.ac.uk/pdbe/entry/pdb/2f8a?activeTab=ligands, accessed on 20 June 2026). In this study, we determined that our ligand EA interacts with these regions of GSH-Px in a similar manner. It has also been observed to form an H-bond in the same way as ASP144 and ARG179. EA exhibited favorable binding affinity toward GSH-Px in the micromolar range.

Although molecular docking cannot directly demonstrate enzyme activation or inhibition under physiological conditions, the observed binding interactions provide complementary structural evidence that supports the biochemical findings of the present study. The ability of EA to interact with functionally important amino acid residues in SOD, CAT, and GSH-Px suggests that these antioxidant enzymes may be potential molecular targets that help maintain cellular redox homeostasis during chronic stocking-density stress. Therefore, the docking analyses should be interpreted as mechanistic support for the in vivo observations rather than as direct evidence of enzymatic regulation. The consistency between the docking results and the enhanced antioxidant enzyme activities observed in vivo further strengthens the hypothesis that dietary EA helps preserve antioxidant defense capacity and physiological resilience under intensive aquaculture conditions.

## 5. Conclusions

Collectively, these findings demonstrate that dietary EA supplementation is not only an effective antioxidant supplement but also a promising nutritional strategy for improving physiological resilience under chronic husbandry stress. Unlike previous studies that focused primarily on chemically induced oxidative stress, the present study shows that EA simultaneously improves growth performance, immune competence, antioxidant defense, and disease resistance under intensive aquaculture conditions, providing a more comprehensive understanding of its biological role in maintaining fish health. These findings highlight the potential of EA as a functional feed additive to enhance fish welfare and promote sustainable aquaculture production. Although tissue EA concentrations were not determined in the present study, future investigations incorporating tissue distribution and pharmacokinetic analyses would further strengthen the mechanistic understanding of the biological effects of ellagic acid. Future studies should further elucidate the molecular signaling pathways underlying these protective effects and evaluate the long-term efficacy of EA throughout the production cycle, including reproductive performance, under commercial farming conditions with variable environmental challenges, such as fluctuations in water temperature and microbial load. In addition, comparative studies involving other commercially important aquaculture species would help determine whether the beneficial effects of dietary EA are species-specific or broadly applicable across aquaculture systems.

## 6. Study Limitations

The present study has several limitations that should be considered when interpreting the findings. First, the experiment was conducted under controlled laboratory conditions, which may not fully reflect the environmental variability encountered in commercial aquaculture systems, including fluctuations in water temperature, microbial load, and other husbandry-related factors. Second, although the 12-week feeding trial was sufficient to evaluate the biological effects of dietary ellagic acid under the present experimental conditions, longer-term studies covering the entire production cycle, including reproductive performance, are needed to assess its sustained efficacy. Third, the present study focused primarily on physiological, immunological, antioxidant, and molecular docking responses. Therefore, additional investigations incorporating gut microbiota profiling as well as transcriptomic and metabolomic analyses would provide deeper mechanistic insight into the pathways underlying the observed biological effects. Finally, residual ethanol concentration in the experimental diets was not analytically determined following feed preparation. Although all dietary treatments, including the control diet, were prepared using identical procedures, future studies should verify residual solvent levels analytically to further strengthen methodological characterization.

## Figures and Tables

**Table 1:** Effects of dietary EA (EA; 0, 50, or 100 mg/kg) supplementation on hematological parameters in carp reared under different stocking densities (SD; 25 kg/m^3^ and 100 kg/m^3^).

Stocking Density (SD)	Ellagic Acid (EA)	RBC, ×10^6^	Hb, g/dL	Ht, %	MCV, µm^3^	MCH, pg	MCHC, %
25 kg/m^3^		1.58 ± 0.49	7.18 ± 0.69	36.35 ± 5.01	246.15 ± 56.03	49.12 ± 12.14	19.93 ± 1.73
100 kg/m^3^		1.23 ± 0.42	6.30 ± 0.54	23.85 ± 6.07	202.36 ± 43.44	56.61 ± 17.97	27.96 ± 6.72
	0 mg/kg	1.23 ± 0.43 ^B^	6.46 ± 0.94 ^B^	25.67 ± 8.13 ^C^	215.73 ± 60.39 ^B^	57.38 ± 17.00 ^A^	27.51 ± 8.52 ^A^
	50 mg/kg	1.45 ± 0.48 ^A^	6.79 ± 0.62 ^A^	30.26 ± 7.72 ^B^	220.00 ± 50.09 ^AB^	51.63 ± 15.74 ^AB^	23.45 ± 4.14 ^B^
	100 mg/kg	1.53 ± 0.50 ^A^	6.98 ± 0.59 ^A^	34.37 ± 6.95 ^A^	237.04 ± 51.29 ^A^	49.58 ± 13.49 ^B^	20.88 ± 3.05 ^C^
25 kg/m^3^	0 mg/kg	1.39 ± 0.43	7.07 ± 0.94 ^ab^	32.38 ± 3.08	251.93 ± 65.13 ^a^	54.39 ± 12.12	21.77 ± 0.87 ^cd^
	50 mg/kg	1.62 ± 0.47	7.24 ± 0.49 ^a^	36.72 ± 4.67	239.28 ± 45.55 ^a^	47.98 ± 11.74	19.87 ± 1.22 ^de^
	100 mg/kg	1.73 ± 0.53	7.23 ± 0.59 ^a^	39.94 ± 3.97	247.23 ± 56.85 ^a^	44.98 ± 10.93	18.15 ± 0.40 ^e^
100 kg/m^3^	0 mg/kg	1.08 ± 0.38	5.84 ± 0.36 ^d^	18.96 ± 5.65	179.53 ± 21.60 ^c^	60.36 ± 20.55	33.25 ± 8.88 ^a^
	50 mg/kg	1.28 ± 0.44	6.34 ± 0.35 ^c^	23.79 ± 3.59	200.72 ± 47.53 ^bc^	55.28 ± 18.41	27.02 ± 2.63 ^b^
	100 mg/kg	1.34 ± 0.41	6.72 ± 0.49 ^bc^	28.80 ± 4.28	226.84 ± 43.64 ^ab^	54.18 ± 14.38	23.60 ± 1.84 ^c^
------------------*p* values-----------------							
	SD	<0.001	<0.001	<0.001	<0.001	0.001	<0.001
	EA	0.001	<0.001	<0.001	0.042	0.015	<0.001
	SD × EA	0.878	0.003	0.312	0.013	0.842	<0.001
	Contrast						
EA effect for SD 25 kg/m^3^	Linear	0.007	0.372	<0.001	0.748	0.002	<0.001
	Quadratic	0.586	0.568	0.525	0.416	0.513	0.666
EA effect for SD 100 kg/m^3^	Linear	0.017	<0.001	<0.001	<0.001	0.186	<0.001
	Quadratic	0.464	0.524	0.933	0.779	0.621	0.251

RBC: Red blood cell; Hb: Hemoglobin; Ht: Hematocrit; MCV: Mean corpuscular volume; MCH: Mean corpuscular hemoglobin; MCHC: Mean corpuscular hemoglobin concentration. ^A,B,C^ Differences between means indicated by different capital letters in the same column are statistically significant for EA doses (*p* < 0.05). ^a,b,c,d,e^ Differences between means indicated by different letters in the same column are statistically significant for interaction between SD and EA (SD × EA; *p* < 0.05). Data are presented as mean and standard deviation.

**Table 2:** Effects of dietary EA (EA; 0, 50, or 100 mg/kg) supplementation on white blood cell counts, nitroblue tetrazolium assay, phagocytic activity, phagocytic index, total protein level, and total immunoglobulin M level in carp reared under different stocking densities (SD; 25 kg/m^3^ and 100 kg/m^3^).

Stocking Density (SD)	Ellagic Acid (EA)	WBC, ×10^3^	NBT, mg/mL	PA, %	PI	TP, mg/mL	IgM, mg/mL
25 kg/m^3^		40.97 ± 5.59	1.32 ± 0.33	37.67 ± 6.18	3.67 ± 0.64	31.60 ± 6.35	16.61 ± 3.76
100 kg/m^3^		28.53 ± 4.08	0.97 ± 0.21	26.56 ± 3.93	2.80 ± 0.38	21.28 ± 3.78	9.41 ± 2.33
	0 mg/kg	30.71 ± 6.91 ^C^	0.99 ± 0.29 ^B^	27.82 ± 5.65 ^C^	2.92 ± 0.53 ^C^	22.45 ± 5.67 ^C^	10.75 ± 4.21 ^C^
	50 mg/kg	35.15 ± 7.62 ^B^	1.19 ± 0.32 ^A^	32.52 ± 7.06 ^B^	3.27 ± 0.66 ^B^	26.63 ± 6.38 ^B^	13.05 ± 4.26 ^B^
	100 mg/kg	38.39 ± 7.35 ^A^	1.26 ± 0.31 ^A^	36.02 ± 7.67 ^A^	3.52 ± 0.72 ^A^	30.23 ± 7.74 ^A^	15.22 ± 4.80 ^A^
25 kg/m^3^	0 mg/kg	36.37 ± 4.82	1.13 ± 0.28	32.05 ± 4.25 ^c^	3.23 ± 0.50 ^c^	26.49 ± 4.06 ^c^	13.99 ± 3.25
	50 mg/kg	41.92 ± 3.81	1.38 ± 0.30	38.33 ± 4.75 ^b^	3.72 ± 0.61 ^b^	31.75 ± 4.88 ^b^	16.57 ± 2.85
	100 mg/kg	44.61 ± 4.64	1.45 ± 0.32	42.63 ± 4.27 ^a^	4.07 ± 0.51 ^a^	36.55 ± 5.57 ^a^	19.25 ± 3.22
100 kg/m^3^	0 mg/kg	25.05 ± 2.76	0.85 ± 0.22	23.58 ± 3.12 ^e^	2.60 ± 0.33 ^e^	18.40 ± 3.89 ^e^	7.51 ± 1.92
	50 mg/kg	28.38 ± 2.95	0.99 ± 0.20	26.71 ± 3.01 ^d^	2.82 ± 0.32 ^de^	21.52 ± 2.20 ^d^	9.52 ± 1.73
	100 mg/kg	32.17 ± 2.89	1.06 ± 0.15	29.40 ± 3.30 ^cd^	2.98 ± 0.40 ^cd^	23.91 ± 2.85 ^cd^	11.19 ± 1.70
------------------*p*-values-----------------							
	SD	<0.001	<0.001	<0.001	<0.001	<0.001	<0.001
	EA	<0.001	<0.001	<0.001	<0.001	<0.001	<0.001
	SD × EA	0.268	0.358	0.003	0.021	0.010	0.229
	Contrast						
EA effect for SD 25 kg/m^3^	Linear	<0.001	<0.001	<0.001	<0.001	<0.001	<0.001
	Quadratic	0.153	0.161	0.323	0.598	0.833	0.939
EA effect for SD 100 kg/m^3^	Linear	<0.001	<0.001	<0.001	<0.001	<0.001	<0.001
	Quadratic	0.721	0.407	0.759	0.670	0.602	0.669

WBC: White blood cell counts; NBT: Nitroblue tetrazolium assay; PA: Phagocytic activity; PI: Phagocytic index; TP: Total protein level; IgM: Total immunoglobulin M level. ^A,B,C^ Differences between means indicated by different capital letters in the same column are statistically significant for EA doses (*p* < 0.05). ^a,b,c,d,e^ Differences between means indicated by different letters in the same column are statistically significant for interaction between SD and EA (SD × EA; *p* < 0.05). Data are presented as mean and standard deviation.

**Table 3:** Effects of dietary EA (EA; 0, 50, or 100 mg/kg) supplementation on serum bactericidal activity, lysozyme activity, and myeloperoxidase activity in carp reared under different stocking densities (SD; 25 kg/m^3^ and 100 kg/m^3^).

Stocking Density (SD)	Ellagic Acid (EA)	BA, % cfu/Control	LYZ, U/mL	MPO, Optic Density
25 kg/m^3^		44.86 ± 12.12	140.45 ± 25.47	0.86 ± 0.20
100 kg/m^3^		24.20 ± 4.61	86.93 ± 15.97	0.50 ± 0.11
	0 mg/kg	25.96 ± 6.87 ^C^	92.79 ± 23.67 ^C^	0.54 ± 0.16 ^C^
	50 mg/kg	34.54 ± 11.20 ^B^	114.78 ± 29.20 ^B^	0.69 ± 0.22 ^B^
	100 mg/kg	43.09 ± 16.04 ^A^	133.50 ± 35.91 ^A^	0.81 ± 0.27 ^A^
25 kg/m^3^	0 mg/kg	31.60 ± 3.87 ^c^	113.38 ± 12.09 ^c^	0.67 ± 0.09 ^c^
	50 mg/kg	44.80 ± 5.36 ^b^	141.59 ± 13.01 ^b^	0.88 ± 0.14 ^b^
	100 mg/kg	58.18 ± 6.43 ^a^	166.37 ± 14.86 ^a^	1.04 ± 0.17 ^a^
100 kg/m^3^	0 mg/kg	20.31 ± 3.90 ^f^	72.19 ± 10.79 ^f^	0.41 ± 0.09 ^e^
	50 mg/kg	24.27 ± 2.90 ^e^	87.97 ± 8.88 ^e^	0.50 ± 0.09 ^d^
	100 mg/kg	28.00 ± 3.30 ^d^	100.62 ± 12.94 ^d^	0.58 ± 0.09 ^d^
------------------*p*-values-----------------				
	SD	<0.001	<0.001	<0.001
	EA	<0.001	<0.001	<0.001
	SD × EA	<0.001	<0.001	<0.001
	Contrast			
EA effect for SD 25 kg/m^3^	Linear	<0.001	<0.001	<0.001
	Quadratic	0.940	0.568	0.381
EA effect for SD 100 kg/m^3^	Linear	<0.001	<0.001	<0.001
	Quadratic	0.880	0.527	0.773

BA: Serum bactericidal activity; LYZ: Lysozyme activity; MPO: Myeloperoxidase activity. ^A,B,C^ Differences between means indicated by different capital letters in the same column are statistically significant for EA doses (*p* < 0.05). ^a,b,c,d,e,f^ Differences between means indicated by different letters in the same column are statistically significant for interaction between SD and EA (SD × EA; *p* < 0.05). Data are presented as mean and standard deviation.

**Table 4:** Molecular docking scores, interaction types, and estimated inhibition constants of EA on SOD, CAT, and GSH-Px.

Macromolecule/Pdb ID	Auto Dock Results			
	Interacting Residues	Interaction Types	Estimated Inhibition Constant, Ki	Best Docking Score
SOD/3LSU	C: ALA183 C: ASP184 D: THR33	Hydrogen Bond Hydrogen Bond Hydrogen Bond	14.34 µM	−6.61
CAT/1DGB	PHE198 PHE198 ARG203 TYR215	Hydrogen Bond Pi-Pi Interaction Hydrogen Bond Hydrogen Bond	19.38 µM	−6.43
GSH-Px/2F8A	A: ASP144 A: TRP160 A: ARG179	Hydrogen Bond Hydrogen Bond Hydrogen Bond	879.85 µM	−4.17

µM: micromolar; docking score: kcal/mol.

## Data Availability

The data presented in this study are available from the corresponding author upon reasonable request.
